# Reproducibility of freehand vs. foam cast as well as the intrarater reliability of foam cast ultrasound scans assessing the muscle architecture and tissue organization of the gastrocnemius medialis and vastus lateralis muscles

**DOI:** 10.3389/fspor.2024.1383411

**Published:** 2024-05-02

**Authors:** Melanie Lesinski, Gregory Bashford, Adrian Markov, Lucie Risch, Michael Cassel

**Affiliations:** ^1^ Division of Training and Movement Sciences, Research Focus Cognition Sciences, University of Potsdam, Potsdam, Germany; ^2^ Department of Biological Systems Engineering, University of Nebraska, Lincoln, NE, United States; ^3^ Department of Sports Medicine, University Outpatient Clinic, University of Potsdam, Potsdam, Germany

**Keywords:** sonography, foam cast, spatial frequency analysis, leg muscles, muscle tissue organization

## Abstract

**Background:**

This study compares the reproducibility of freehand (FH) vs. foam cast (FC) scans and investigates the intrarater reliability of the ultrasound FC muscle architecture and tissue organization measurements of the gastrocnemius medialis (GM) and vastus lateralis (VL) muscles with fixed and repositioning FC scans.

**Methods:**

Thirteen young adults (22 ± 3 years) underwent repeated sagittal B-mode ultrasound measurements of GM and VL. FH, FC, and repositioned FC scans were conducted. Muscle architecture measurements included muscle thickness (MT), pennation angle (PA), and fascicle length (FL). Spatial frequency analysis assessed muscle tissue organization.

**Results:**

MT decreased from 2.1 to 1.8 cm in GM and from 2.4 to 2.2 cm in VL with the FC compared with the FH. Reproducibility between the FH and the FC showed poor to good intraclass correlation coefficients (ICCs) for MT (0.46–0.77) and PA (0.09–0.86) as well as poor to moderate ICCs for FL (0.41), with very low to moderate test–retest variability (TRV) (4%–18%). Tissue organization indicated low to good ICCs (0.21–0.80) with low to moderate TRV (4%–19.5%). The re-scanning results of fixed FC indicated excellent ICCs for MT (0.95–0.996), good for PA (0.77–0.90), and moderate for FL (0.73–0.76), with low TRV (5%–10%) for both muscles. Tissue organization displayed moderate to good ICCs (0.61–0.87) with very low to low TRV (4%–9%). For repositioned FC scans in GM and VL, MT showed good to excellent ICCs (0.86–0.98) with very low to low TRV (2%–8%). PA and FL demonstrated moderate to good ICCs (0.57–0.75), with very low to moderate TRV (2%–13%). Tissue organization revealed ICCs ranging from poor to good (0.13–0.87) for both muscles, with low to moderate TRV (5%–18%).

**Conclusion:**

The FC systematically reduced MT by 2–3 mm. Furthermore, reproducibility revealed low ICCs and high data variability for several muscle architecture and tissue organization parameters. Thus, switching methods within a single study is not recommended. Nevertheless, FC ultrasound scans demonstrated excellent intrarater reliability for assessing MT. In the case of fixed FC scans particularly, moderate to excellent ICCs were observed for all muscle architecture and tissue organization parameters, accompanied by very low to low variability. Therefore, FC scans are recommended for investigating acute effects on muscle architecture and tissue organization when the FC remains on the leg throughout the period of measurements.

## Background

Muscle architecture [i.e., muscle thickness (MT), fascicle length (FL), and fascicle pennation angle (PA)] affects the force-producing capabilities of human skeletal muscles (1). This tissue architecture also holds potential for quantifying adaptations or changes in human skeletal muscles resulting from training, injury, or pathology. Recently, not only the muscle architecture but also the muscle tissue organization (2, 3) has been quantified for acute and chronic adaptations in muscles indicating the overall tissue integrity at the micromorphological level. To analyze muscle tissue organization, spatial frequency analysis (SFA) can be conducted, following the methodology outlined by Bashford et al. (4, 5). SFA is a quantitative ultrasound method that examines the anisotropic B-mode speckle pattern produced by a tissue type in the spatial frequency domain. In essence, this technique involves manually delineating a polygonal region of interest (ROI) on an image, within which smaller subregions referred to as “kernels” are examined in the spatial frequency domain. For each kernel within the ROI, parameters from the fast Fourier transform (FFT)-derived spatial frequency spectral estimate are extracted. B-mode ultrasound imaging has become a popular method of investigating human skeletal muscle architecture and tissue organization *in vivo* because of its more affordable, portable, safe and non-invasive nature (6).

To obtain meaningful information on muscle architecture and tissue organization from ultrasound imaging, standardization of image acquisition [i.e., probe placement, probe rotation, probe orientation (e.g., angle), and probe pressure] is necessary (7–9). In a previous report (5), the inter- and intrarater reliability of freehand (FH) ultrasound measurements assessing the skeletal muscle architecture and tissue organization of the gastrocnemius medialis (GM) and vastus lateralis (VL) muscle was assessed. The findings demonstrated poor to excellent intraclass correlation coefficients (ICCs: 0.41–0.99) with very low to moderate test–retest variability (TRV, 3%–11%) for the inter- and intrarater reliability of muscle architecture as well as poor to good ICCs (0.29–0.87) with very low to moderate TRV (3%–14%). When the probe is manually held, there may be slight differences in probe rotation and orientation between different investigators and also between different measurements of the same investigators, all of which affects reliability. As B-mode ultrasound imaging is normally limited to a two-dimensional view, standardization of the visualization plane (probe rotation and orientation) of the three-dimensional muscle structure is crucial. By varying probe rotation and probe orientation slightly (e.g., misalignment of perpendicular probe orientation), PA and FL can be significantly under- or overestimated (7, 8). Klimstra et al. (8), for instance, highlighted that changes in probe rotation and orientation can result in a 12% difference in the reported PA. Therefore, a special foam cast (FC) may bring advantages for standardized acquisition of ultrasound scans (e.g., probe orientation). In addition, since FH ultrasound scans are not always possible (e.g., during specific testing positions and dynamic/functional tests such as jumping and running tasks), alternative measurement conditions such as a special FC can become necessary.

Although some studies have utilized foam casts when examining the reliability of ultrasound scans for assessing muscle architecture parameters (10), only one study to date (11) has directly compared the reproducibility of FH vs. FC scans for muscle architecture, and no study has been conducted for tissue organization parameters. Furthermore, it remains to be tested to what extent ultrasound measurements using the FC method yield reliable data when the FC remains on the leg between measurements (fixed FC scans) or when the FC is detached and reattached between measurements (repositioning FC scans). Thus, the reliability of FC ultrasound scans requires an evaluation to provide guidance for achieving optimal standardization of ultrasound image acquisition.

Hence, the aim of this study was to compare the method of FH vs. FC scans and investigate the intrarater reliability of ultrasound FC muscle architecture, specifically the tissue organization measurements of GM and VL with fixed (the FC remaining on the leg) and repositioning (the FC detached and reattached between measurements) FC scans.

## Materials and methods

### Study design

**Figure 1:**
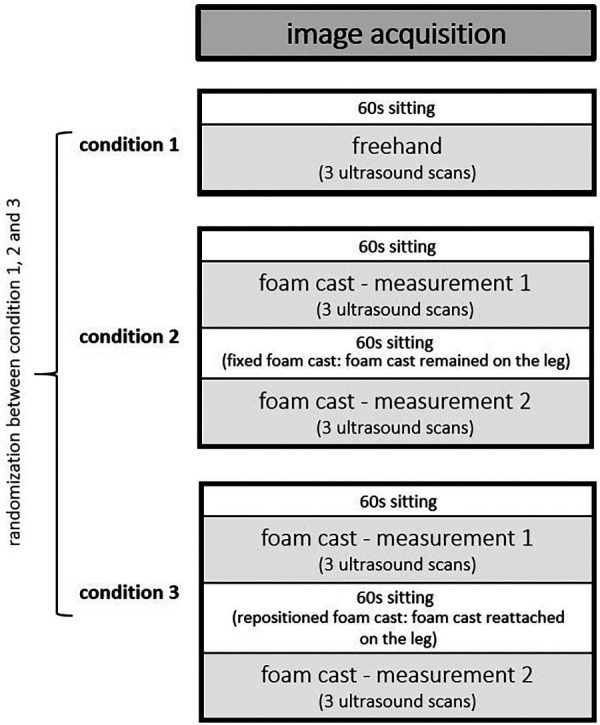
Randomized ultrasound image acquisition.

A single-group, repeated-measures study design was conducted to compare (1) the reproducibility of FH vs. FC scans (i.e., condition 2, measurement 1; Figure 1) and the intrarater reliability of the ultrasound FC muscle architecture and tissue organization measurements of GM and VL with (2) fixed (i.e., the FC remaining on the leg) and (3) repositioning (i.e., the FC detached and reattached between measurements) FC scans at rest.

**Figure 2:**
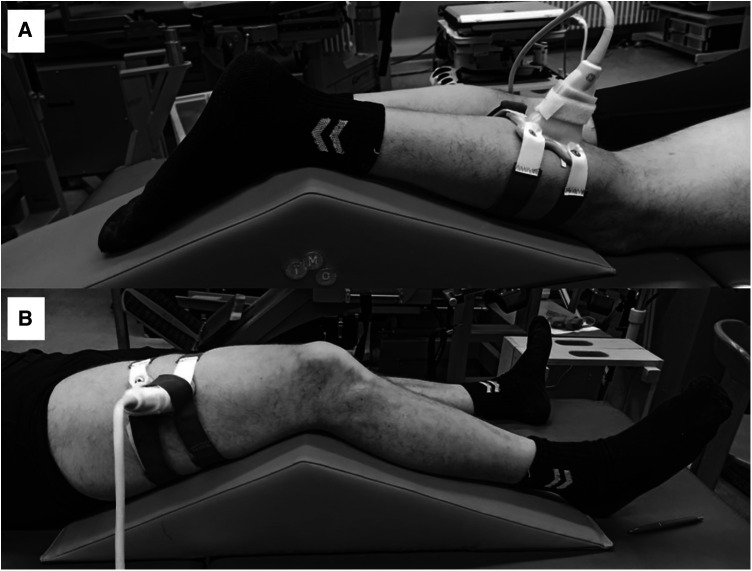
Ultrasound foam cast scans of the gastrocnemius medialis (**A**) and vastus lateralis muscles (**B**).

All assessments were conducted by the same investigator with one year of sonographic experience with guidance from a sports orthopedic physician with 15 years of experience in musculoskeletal ultrasound. During FH scans, the investigator held the probe manually at a predefined location. For the FC scans, a custom-made FC was fixed on the same predefined location on the leg with Velcro straps (Figure 2). The GM location was defined at a point one-third of the distance between the popliteal crease (tendon of the semitendinosus muscle) and the medial malleolus. The VL location was defined half way between the middle of the patella and the greater trochanter (12).

After image acquisitions were completed for one condition, the participant sat on the examination table for 60 s (13), before laying back down to get measured again for the next condition (Figure 1). The measurements were performed in randomized order (Figure 1). For each condition, three scans of the GM and VL muscle were obtained at the same location, resulting in a total of 156 images for each muscle.

### Participants

Seven male and six female healthy young adults aged 19–30 years (22 ± 3 years, body height: 174 ± 8 cm, body mass: 69.7 ± 10.1 kg, body fat: 15.7% ± 5.5%) volunteered to participate in this study. The participants were physically active adults (i.e., sports students) who attend weekly practical sports courses as part of their studies and regularly engage in various types of sports training such as volleyball, gymnastics, soccer, fitness, athletics, tennis, dancing, and kickboxing in their free time. Sample size calculations were conducted following those stipulated by Borg et al. (14) and Zou (15). Exclusion criteria were defined *a priori* as any musculoskeletal, neurological, and/or orthopedic disorders in the lower extremities that occurred within the last 6 months prior to the start of the study. Written informed consent was obtained from all participants prior to their inclusion in the study. The study was approved by the local ethics committee. All experiments were conducted according to the latest version of the Declaration of Helsinki (16).

### Measurement procedure

Initially, the measurement locations of GM and VL were marked on the dominant (right) leg of all participants. To recover the marked measurement location in the ultrasound images, a thin strip of echoabsorptive tape was placed 1.5 cm proximal to the previously marked measurement location (5). GM and VL were assessed under the resting condition. The participants lay prone on an examination table with the right leg supported on an inclined foam wedge (ankle 40° extended) to assess the right GM muscle (Figure 2). For assessing the right VL muscle, participants lay supine on an examination table with the right leg supported on an inclined foam wedge (knee 25° flexed; Figure 2). Longitudinal ultrasound scans (Vivid q; GE Healthcare, Tirat Carmel, Israel) of the GM and VL muscle belly were conducted by using a linear ultrasound transducer (6 cm probe width, 4–13 MHz). The preset was standardized (frequency: 11 MHz; depth: 4.5 cm; gain: 38%; dynamic range: 102; foci for GM: 1.2 and 2.5 cm; foci for VL: 1.625 and 3.125 cm) and kept constant for all image acquisitions.

### Data analysis

To ensure blinded image evaluation, all saved and transferred images were assigned random alphanumeric codes during the analysis. ImageJ software (National Institutes of Health, Bethesda, MD, USA, version: 1.53s) was used to analyze the skeletal muscle architecture (i.e., MT, inferior/superior PA, FL). In terms of muscle architecture, MT was measured as the distance between the upper and the lower aponeuroses at the predefined position (1.5 cm distal from the marking tape). Superior PA was measured as the angle between the upper aponeurosis and the fascicle. Inferior PA was measured as the angle between the lower aponeurosis and the fascicle. The FL was calculated from the visible FL (FL1) plus the calculated FL (FL2), according to Baudry et al. (17). Subsequently, all ultrasound images were imported into MATLAB (MathWorks, USA, R2016a) to conduct SFA to analyze muscle tissue organization as described by Bashford et al. (4) and recently updated (5). The ROI was selected in a standardized manner by measuring a 1 cm wide rectangular area of the GM and VL from the upper to the lower aponeuroses at the measurement location (5). The six resulting spatial frequency parameters were peak spatial frequency radius (PSFR), peak −6 dB width (P6), PSFR/P6 (Q6), normalized peak value of amplitude spectrum (Amax), power within peak (PWP), and peak power percent (PPP) (5). A graphical representation of all muscle architecture parameters, as well as a detailed description of all SFA parameters, can be found in Lesinski et al. (5).

### Statistical analysis

The mean of the three scans for each condition was used for statistical analyses (Figure 1). The data were normally distributed (Shapiro–Wilk test) and presented descriptively by mean ± standard deviation (SD). Both reproducibility and reliability were quantitatively assessed by using the paired *t*-test, the ICC [2.1 (18)] with its respective 95% confidence interval (CI), the test–retest variability ($\mathrm{TRV}\text{\%}=\left|\frac{\mathrm{difference}}{\mathrm{mean}}\right|\times \;100\text{\%}$), the standard error of measurement [$\mathrm{SEM}=\mathrm{SD}\times \sqrt{\left(1-r\right)}$ (19, 20)], and SEM% ($\frac{\mathrm{SEM}\;\times \;100\text{\%}}{\mathrm{mean}}$). The level of ICC values was defined as poor (ICC < 0.5), moderate (0.5 ≤ ICC < 0.75), good (0.75 ≤ ICC ≤ 0.9), or excellent (ICC > 0.9) (18). The level of variability was defined as very low (TRV% < 5%), low (5% ≤ TRV% < 10%), moderate (10% ≤ TRV% < 20%), and high (TRV% > 20%), as proposed by us in Lesinski et al. (5). Furthermore, Bland-Altman analyses were conducted to determine the Bias (mean difference) and the 95% limits of agreement (LoA). To check the validity of LoA data, homoscedasticity was examined (19, 21, 22) by applying the Breusch–Pagan test. The results were considered statistically significant at *p* < 0.05. The calculations of the paired *t*-test, the ICCs, and the Breusch–Pagan test were performed with IBM SPSS Statistics Version 26.0 (IBM Corporation, Armonk, NY, USA). The calculations of bias, LoA, SEM, and SEM% were performed in Microsoft Excel 2016 (Microsoft Corporation, Redmond, WA, USA).

## Results

### Reproducibility between freehand vs. foam cast scans

**Figure 3:**
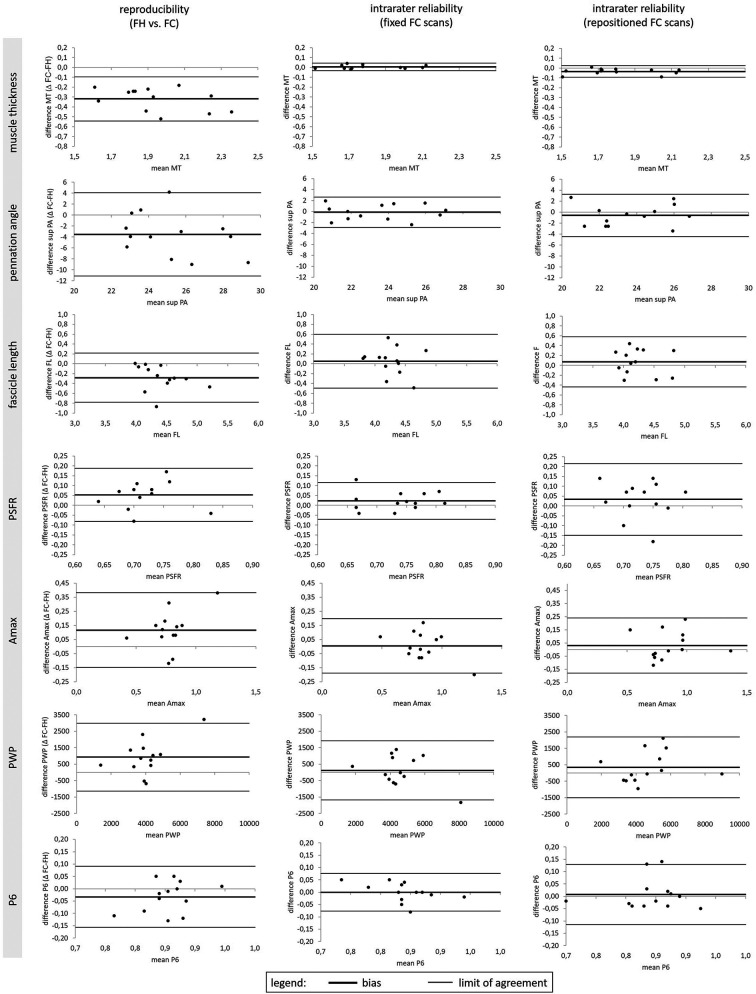
Bland–Altman plots for Gastrocnemius medialis muscle thickness (MT), superior pennation angle (sup. PA), fascicle length (FL), Peak Spatial Frequency Radius (PSFR), Amax, PWP and P6 for the method comparison reproducibility between freehand (FH) vs. foam cast (FC) measurements as well as intra-rater reliability of ultrasound fixed and repositioned FC measurements.

The mean values ± SD for the assessed GM and VL muscle architecture and tissue organization by using FH vs. FC scans are presented in Table 1. Reproducibility between the FH and the FC for GM and VL is shown in Table 2 and Figure 3. A comparison of FH vs. FC ultrasound images indicated only poor to good ICCs for GM (0.09–0.86) and VL (0.27–0.77) muscle architectures, with very low to moderate TRV (4.4%–17.8%). In terms of muscle tissue organization parameters, poor to moderate ICC values were found for GM (0.21–0.61), compared with moderate to good ICC values for VL (0.53–0.80), with very low to moderate TRV (3.5–19.5%), except high TRV (27.3%) for PWP (for GM only). Particularly for GM, a paired *t*-test revealed several significant differences between FH and FC scans of the same parameters (i.e., MT, superior PA, FL, Q6, Amax, and PWP).

**Table 1:** Gastrocnemius medialis and vastus lateralis muscle architecture and tissue organization.

Parameter	Gastrocnemius medialis		Vastus lateralis	
	Freehand	Foam cast	Freehand	Foam cast
Muscle thickness (cm)	2.10 ± 0.26	1.79 ± 0.21	2.41 ± 0.35	2.23 ± 0.39
Superior pennation angle (°)	27.0 ± 3.5	23.4 ± 2.4	12.8 ± 2.9	13.1 ± 2.0
Inferior pennation angle (°)	28.6 ± 3.0	28.3 ± 3.1	13.2 ± 2.4	13.9 ± 2.1
Fascicle length (cm)	4.55 ± 0.40	4.27 ± 0.32	10.81 ± 2.20	9.45 ± 1.42
PSFR (mm^−1^)	0.69 ± 0.06	0.75 ± 0.06	0.86 ± 0.11	0.89 ± 0.11
P6 (mm^−1^)	0.87 ± 0.04	0.84 ± 0.06	0.80 ± 0.03	0.81 ± 0.04
Q6	0.98 ± 0.09	1.11 ± 0.12	1.28 ± 0.13	1.30 ± 0.12
Amax (B/sample)	0.72 ± 0.15	0.84 ± 0.21	0.81 ± 0.15	0.83 ± 0.14
PWP (B^2^)	3,567 ± 1,126	4,498 ± 1,655	4,187 ± 1,424	4,365 ± 1,229
PPP (%)	69.5 ± 3.5	68.0 ± 4.8	66.3 ± 4.1	66.6 ± 3.4

**Table 2:** Reproducibility of freehand vs. foam cast scans for the gastrocnemius medialis and vastus lateralis muscle architecture and tissue organization.

Muscle	Parameter	*t*-test (*p*)	ICC (95% CI)	Bias ± LoA	TRV, %	SEM	SEM, %
Gastrocnemius medialis	Muscle thickness (cm)	<0.001	0.46 (−0.05–0.84)	−0.32 ± 0.22	16.3	0.17	8.6
	Superior pennation angle (°)	0.003	0.09 (−0.17–0.47)	−3.5 ± 7.6	17.1	2.2	8.6
	Inferior pennation angle (°)	0.75	0.86 (0.60–0.96)	−0.2 ± 3.3	4.4	1.1	3.8
	Fascicle length (cm)	<0.001	0.41 (−0.11–0.78)	−0.28 ± 0.50	6.3	0.26	5.9
	PSFR (mm^−1^)	0.07	0.21 (−0.18–0.62)	0.05 ± 0.13	10.3	0.04	5.7
	P6 (mm^−1^)	0.10	0.27(−0.21–0.68)	−0.03 ± 0.12	6.5	0.04	4.2
	Q6	<0.001	0.35 (−0.12–0.74)	0.13 ± 0.18	12.8	0.07	7.2
	Amax (B/sample)	0.01	0.60 (0.03–0.87)	0.12 ± 0.27	18.4	0.10	13.4
	PWP (B^2^)	0.01	0.61 (0.02–0.87)	931 ± 2,066	27.3	826	20.5
	PPP (%)	0.22	0.51 (0.001–0.82)	−1.5 ± 8.2	5.4	2.6	3.8
Vastus lateralis	Muscle thickness (cm)	0.01	0.77 (0.23–0.93)	−0.18 ± 0.40	9.5	0.17	7.4
	Superior pennation angle (°)	0.58	0.53 (0.02–0.84)	0.3 ± 4.9	14.4	1.5	11.6
	Inferior pennation angle (°)	0.44	0.27 (−0.30–0.71)	0.7 ± 5.4	16.8	1.5	11.3
	Fascicle length (cm)	0.02	0.41 (−0.07–0.76)	−1.36 ± 3.59	17.8	1.23	12.2
	PSFR (mm^−1^)	0.27	0.73 (0.34–0.91)	0.03 ± 0.16	8.8	0.05	6.1
	P6 (mm^−1^)	0.30	0.53 (0.02–0.83)	0.01 ± 0.07	3.5	0.02	2.4
	Q6	0.49	0.69 (0.25–0.89)	0.02 ± 0.20	6.8	0.06	4.9
	Amax (B/sample)	0.43	0.80 (0.47–0.93)	0.02 ± 0.19	10.2	0.06	7.7
	PWP (B^2^)	0.51	0.76 (0.39–0.92)	177 ± 1,835	19.5	610	14.3
	PPP (%)	0.70	0.65 (0.17–0.88)	0.3 ± 6.3	4.1	2.0	3.0

### Intrarater reliability of foam cast scans

#### Fixed positioning

The intrarater reliability (fixed positioning) for GM and VL is presented in Table 3 and Figure 3. For both muscles, the ICC showed excellent values for MT (0.95–0.996), good values for PA (0.77–0.90), and moderate values for FL (0.64–0.73). TRV was less than 6% for GM and less than 11% for the VL muscle architecture. With regard to muscle tissue organization, analyses indicated moderate to good ICC values (0.59–0.87), with very low to low TRV (2.7%–9.3%), except moderate variability for PWP (TRV%: 14.6%–15.9%). The paired *t*-test revealed no significant differences between fixed FC scans of the same parameters.

**Table 3:** Intrarater reliability (fixed positioning) for the gastrocnemius medialis and vastus lateralis muscle architecture and tissue organization.

Muscle	Parameter	*t*-test (*p*)	ICC (95% CI)	Bias ± LoA	TRV, %	SEM	SEM, %
Gastrocnemius medialis	Muscle thickness (cm)	0.22	0.996 (0.99–0.999)	0.01 ± 0.04	1.0	0.01	0.7
	Superior pennation angle (°)	0.72	0.83 (0.54–0.95)	0.1 ± 2.8	5.1	0.9	4.0
	Inferior pennation angle (°)	0.70	0.83 (0.52–0.94)	−0.2 ± 3.7	5.3	1.2	4.3
	Fascicle length (cm)	0.64	0.76 (0.37–0.92)	0.05 ± 0.55	5.0	0.14	3.3
	PSFR (mm^−1^)	0.14	0.64 (0.20–0.87)	0.02 ± 0.09	5.3	0.03	4.4
	P6 (mm^−1^)	0.95	0.73 (0.31–0.91)	0.00 ± 0.08	3.6	0.03	3.0
	Q6	0.20	0.70 (0.29–0.90)	−0.03 ± 0.18	6.4	0.06	5.5
	Amax (B/sample)	0.80	0.87 (0.63–0.96)	−0.02 ± 0.16	9.3	0.06	7.6
	PWP (B^2^)	0.63	0.82 (0.51–0.94)	124 ± 1,796	15.9	602	13.2
	PPP (%)	0.70	0.62 (0.12–0.87)	0.4 ± 7.4	4.2	2.3	3.4
Vastus lateralis	Muscle thickness (cm)	0.54	0.95 (0.85–0.99)	−0.02 ± 0.23	2.7	0.08	3.6
	Superior pennation angle (°)	0.30	0.77 (0.42–0.92)	0.4 ± 2.4	7.8	0.8	6.2
	Inferior pennation angle (°)	0.82	0.90 (0.71–0.97)	0.1 ± 1.9	5.7	0.6	4.6
	Fascicle length (cm)	0.73	0.73 (0.31–0.91)	−0.12 ± 2.50	10.3	0.81	8.6
	PSFR (mm^−1^)	0.30	0.78 (0.43–0.93)	−0.02 ± 0.16	7.9	0.06	6.3
	P6 (mm^−1^)	0.70	0.61 (0.10–0.86)	0.00 ± 0.06	3.1	0.02	2.6
	Q6	0.32	0.59 (0.11–0.85)	0.04 ± 0.26	8.1	0.08	6.4
	Amax (B/sample)	0.36	0.70 (0.26–0.90)	−0.03 ± 0.22	7.8	0.07	8.4
	PWP (B^2^)	0.24	0.67 (0.21–0.89)	−375 ± 2,051	14.6	687	15.0
	PPP (%)	0.36	0.70 (0.25–0.90)	−0.7 ± 5.2	2.7	1.7	2.6

#### Repositioning

The intrarater reliability (repositioning) for GM and VL is presented in Table 4 and Figure 3. Analyses showed an excellent ICC value of MT for GM and good ICC value for VL, with very low to low variability (TRV%: 2.0%–7.7%). Furthermore, the analyses indicated moderate to good ICC values for the PA and FL of both muscles, with very low to low variability for GM (TRV%: 5.3%–7.1%) and low to moderate variability for VL (TRV%: 10.2%–13.0%). With regard to muscle tissue organization, the analyses indicated poor to good ICC values for GM (0.13–0.87) and VL (0.29–0.76), with low to moderate variability (TRV%: 4.7%–17.7%), except high variability for the PWP of VL (TRV%: 28.9%). Except for the MT of the GM, paired *t*-tests revealed no significant differences between repositioned FC scans for the same parameters.

**Table 4:** Intrarater reliability (repositioning) for the gastrocnemius medialis and vastus lateralis muscle architecture and tissue organization.

Muscle	Parameter	*t*-test (*p*)	ICC (95% CI)	Bias ± LoA	TRV, %	SEM	SEM, %
Gastrocnemius medialis	Muscle thickness (cm)	0.001	0.98 (0.70–0.995)	−0.03 ± 0.06	2.0	0.03	1.7
	Superior pennation angle (°)	0.29	0.63 (0.18–0.87)	−0.6 ± 3.8	7.1	1.3	5.3
	Inferior pennation angle (°)	0.99	0.75 (0.35–0.92)	0.1 ± 3.8	5.3	1.3	4.4
	Fascicle length (cm)	0.33	0.70 (0.28–0.90)	0.07 ± 0.51	5.4	0.17	4.0
	PSFR (mm^−1^)	0.24	0.13 (−0.60–0.42)	0.03 ± 0.18	10.7	0.04	6.0
	P6 (mm^−1^)	0.71	0.49 (−0.08–0.81)	0.01 ± 0.12	5.2	0.04	4.6
	Q6	0.15	0.41 (−0.10–0.77)	−0.05 ± 0.24	9.5	0.07	6.9
	Amax (B/sample)	0.33	0.87 (0.64–0.96)	0.03 ± 0.21	10.7	0.07	8.5
	PWP (B^2^)	0.21	0.85 (0.59–0.95)	346 ± 1,854	17.0	660	14.1
	PPP (%)	0.10	0.37 (−0.11–0.74)	2.5 ± 8.6	5.4	2.7	4.0
Vastus lateralis	Muscle thickness (cm)	0.07	0.86 (0.57–0.96)	−0.11 ± 0.38	7.7	0.14	6.3
	Superior pennation angle (°)	0.42	0.66 (0.20–0.88)	−0.4 ± 3.8	13.0	1.2	9.2
	Inferior pennation angle (°)	0.88	0.63 (0.13–0.87)	0.1 ± 4.4	12.8	1.4	10.1
	Fascicle length (cm)	0.36	0.57 (0.07–0.84)	−0.27 ± 2.33	10.2	0.86	8.9
	PSFR (mm^−1^)	0.96	0.76 (0.32–0.92)	0.00 ± 0.18	7.3	0.06	6.3
	P6 (mm^−1^)	0.61	0.33 (−0.27–0.74)	0.01 ± 0.09	4.7	0.03	3.4
	Q6	0.78	0.51 (−0.07–0.82)	0.01 ± 0.29	8.2	0.09	6.7
	Amax (B/sample)	0.82	0.57 (−0.001–0.86)	−0.01 ± 0.33	17.7	0.10	12.6
	PWP (B^2^)	0.81	0.56 (−0.03–0.85)	106 ± 2,908	28.9	899	20.7
	PPP (%)	0.33	0.29 (−0.30–0.72)	−1.6 ± 10.3	6.2	3.0	4.5

## Discussion

The objective of this study was to compare the reproducibility of FH vs. FC scans and investigate the intrarater reliability of the ultrasound FC muscle architecture and tissue organization measurements of the GM and VL muscles with fixed and repositioning FC scans among healthy young adults. The findings demonstrated that the FC systematically reduced GM and VL muscle thicknesses by 2–3 mm. The ICC values ranged from poor to good between the FH and the FC scan measurements of muscle architecture and tissue organization in GM and VL, exhibiting variability of up to 27%, as well as showing significant differences in several parameters (i.e., MT, superior PA, FL, Q6, Amax, and PWP) between FH and FC scans. Consequently, it is not advisable to switch between FH and FC scans within a single study because of the possibility of the occurrence of systematic errors. Nevertheless, FC ultrasound scans demonstrated excellent intrarater reliability for assessing MT in the GM and VL muscles. In the case of fixed FC scans particularly, moderate to excellent ICC values were observed for all muscle architecture and tissue organization parameters, accompanied by very low to low variability, and there were no significant differences between FH and FC parameters. Therefore, fixed FC scans are strongly recommended for investigating acute effects on muscle architecture and tissue organization when the FC remains on the leg throughout the period of measurements.

Previously, only König et al. (11) had investigated the reproducibility between the FH and the FC ultrasound measurements of the GM muscle architecture (i.e., MT, PA, and FL). They found moderate to good ICC values (0.62–0.86) between the two methods. In contrast, our study revealed lower ICC values (0.09–0.86), indicating slightly poorer reproducibility between FH and FC scans. These disparities in ICC values could be attributed to variations in the type and attachment of the FC. Overall, the data indicated a systematic error, manifesting as a smaller MT and shorter FL when using FC scans compared with FH scans. This error might result from a slightly higher probe pressure when attaching the FC using Velcro straps. Based on our findings, we cannot recommend switching between FH and FC scans during ultrasound image acquisition within a single study of a research project or clinical follow-up, as noted previously.

To date, studies have primarily focused on the intrarater reliability of the FH ultrasound measurements of muscle architecture. In a recent study (5), the inter- and intrarater reliability of FH ultrasound measurements assessing muscle architecture and tissue organization in GM and VL was investigated. In terms of intrarater reliability, that study demonstrated excellent ICC values for MT in the GM and VL muscles (0.93–0.97), while PA and FL exhibited only poor to moderate ICC values for GM (0.41–0.58) and good ICC values for VL (0.82–0.90), with very low to low variability. Further, the study showed for GM and VL an SEM% of less than 9, as well as a systematic bias of 0.01–0.02 cm for MT, 0.12–0.33 cm for FL, and between 0.3° and 1.4° for PA. The findings regarding tissue organization parameters indicated moderate to good ICCs (0.63–0.87, except P6), with very low to low variability as well as a SEM% of less than 8 (except for PWP). Compared with FH scans, FC scans may be a valuable tool for enhancing measurement consistency (i.e., probe orientation), as well as reducing operator-related variability and, in turn, enhancing the overall intrarater reliability of ultrasound measurements. This is crucial for ensuring that ultrasound measurements are taken from the same anatomical location before and after interventions or across different subjects. Human operators can introduce variability in ultrasound measurements because of differences in hand pressure, angle, and placement. An FC may reduce this variability, leading to more reliable and reproducible results. Furthermore, in some cases, researchers may be interested in dynamic measurements such as changes in muscle architecture or tissue organization during functional movement or contraction. An FC can provide enhanced stability, enabling accurate assessment in dynamic scenarios. Our results demonstrated excellent ICCs for intrarater reliability for the FC scans of MT in GM and VL. Furthermore, our results indicated moderate to good ICCs for the intrarater reliability of PA and FL in GM and VL. This applies to both when the FC remains attached to the leg between measurements (fixed positioning) and when it is removed and reattached (repositioned). The reliability of fixed vs. repositioning FC scans is a crucial consideration for assessing the extent to which both acute and chronic effects can be evaluated with the FC. When examining long-term effects, the FC must inevitably be repositioned after weeks of intervention, potentially affecting reliability. In acute investigations, ideally, the FC can simply remain in place. Based on our findings, repeated ultrasound measurements using the FC are considered reliable and therefore are recommended, especially for investigating acute effects on muscle architecture where the FC can remain attached to the leg between measurements.

When it comes to assessing the organization of muscle tissue, SFA has recently proven to be a valuable tool (2). It can effectively reveal differences in muscle structure resulting from factors like training interventions. To harness the potential of SFA for this purpose, it is essential to ascertain the reliability of various SFA parameters. This helps to distinguish between measurement inaccuracies and actual training-induced changes. Crawford et al. (13) conducted a comprehensive reliability study focused on hamstring muscles. They considered four key SFA parameters: PSFR, Mmax, Mmax%, and Sum. Crawford et al. (13) revealed excellent ICCs for interrater interpretation error for the extracted SFA parameters (ICC: 0.95–0.98). As a result, they concluded that SFA could serve as an objective method for estimating changes in muscle tissue due to factors such as muscle hypertrophy, swelling, localized edema, or mechanical disruptions of the perimysium. Recent updates to the SFA algorithm have introduced additional parameters (i.e., Amax, PWP, PPP) that are particularly relevant in the context of muscle research. In a recently published study (5), the reliability of these additional parameters was assessed using FH scanning. The findings demonstrated excellent ICCs for interrater interpretation error and moderate to good ICCs for inter- and intrarater reliability, with very low to low variability (except for PWP). Although some studies have utilized foam casts when examining the reliability of ultrasound scans for assessing muscle architecture parameters (10), there have been no studies to date that directly compare the reproducibility of FH vs. FC scans for tissue organization parameters, as well as consider the intrarater reliability of FC ultrasound scans for muscle tissue organization. Our study is the first to examine these aspects. Our results demonstrate reliability comparable to FH ultrasound scans for muscle tissue organization parameters, especially when the FC remains on the leg between the measurements (fixed FC scans). Thus, our results indicate that FC scans exhibit a similar level of reliability as FH scans, while also providing the added advantage of specific testing positions and the ability to conduct dynamic ultrasound measurements. Regardless of whether FH or FC scans are performed, certain SFA parameters (i.e., PWP) exhibit very high TRV in terms of intrarater reliability and should, therefore, be used with caution during interpretations.

One limitation of this study concerns the consecutive execution of all measurements during both the test and the retest sessions within a single day. This experimental design was deliberately chosen to minimize the influence of confounding variables. In addition, no quantitative method was used to control for muscle compression during the FC data collection process. Nevertheless, the data were always collected by the same investigator, who took great care and visually checked the ultrasound images during capturing to ensure that the muscle was not compressed. Furthermore, it is possible that sitting and subsequently lying down between repeated measurements caused a temporary shift of the skin over the muscle. With insufficient time available before the next measurement, the skin may not have returned to its original position above the muscle, leading to imprecise identification of the same analysis spot by the skin marker. Nevertheless, we assessed the images of several participants to compare the distance between specific muscular features in the ultrasound image and the skin marker from repeated measurements, and we observed no noticeable difference in distance. Furthermore, averaging data for the calculation of reliability, instead of evaluating each trial individually, may be viewed as a type of data smoothing, which could potentially mask some of the variabilities present in the data. In addition, the analyses indicated a discrepancy between the ICC and the variability (TRV%). As noted previously, this inconsistency might be attributed to the sensitivity of the ICC to factors such as sample size, the range of the measurement scale, and variance ratios.

## Conclusion

In summary, our main findings were as follows: first, the FH and FC should not be used interchangeably. The FC resulted in a systematically reduced MT (i.e., 2–3 mm) and revealed significant differences between several FH and FC parameters (i.e., MT, superior PA, FL, Q6, Amax, and PWP). In addition, we observed only poor to good ICCs between the FH and the FC measurements of muscle architecture and tissue organization, with variability reaching up to 27%. Second, FC-derived measurements were moderately reliable when the probe was repositioned (e.g., intersession measurement). Third, the FC-derived measurements were highly reliable when the probe was not repositioned (e.g., intrasession measurement). These results indicate that repeated ultrasound measurements using the FC are reliable and therefore strongly recommended when investigating acute effects on muscle architecture and tissue organization, provided that the FC can remain affixed to the leg throughout the period of measurements. This increased reliability may assist researchers performing more advanced (i.e., dynamic) ultrasound measurements.

## Data Availability

The raw data supporting the conclusions of this article will be made available by the authors without undue reservation.
